# Enhanced recovery after caesarean section: an intrathecal morphine dosing study

**DOI:** 10.1186/s13741-025-00555-3

**Published:** 2025-10-14

**Authors:** Rian Crandon, Nicholas Storr, Paula Parker, Ramil Nair, Melissa Pietrobuono, Ian Hughes

**Affiliations:** 1 Townsville University Hospital, Townsville City, QLD Australia; 2 Gold Coast University Hospital, Gold Coast, QLD Australia

## Abstract

**Objective:**

To compare the analgesic and side effect profiles of three intrathecal morphine doses as part of an enhanced recovery protocol in obstetric patients undergoing caesarean section.

**Methods:**

A prospective cohort study including ASA 2 females, over 18 years old having repeat elective caesarean sections. Primary outcomes include total oral morphine equivalents (mg) at 24 and 48 h, proportion of opioid free patients (24 and 48 h), and frequency of ITM-related side-effects. Secondary outcome measures include duration of analgesia, pain scores at 24 h, FAS at 24 h and length of stay.

**Results:**

Five hundred seventy-four patients were divided into 150 mcg (190 patients), 125 mcg (191 patients) and 100 mcg (193 patients) intrathecal morphine groups. Effective analgesia was provided by all doses of ITM with a dose-dependent increase in side-effects. The median opioid consumption at 24 h was 10 mg in the 150 mcg group and 20 mg in the 125 mcg and 100 mcg groups. At 48 h, the median opioid consumption was 30 mg in the 150 mcg group and 45 mg in the 125 mcg and 100 mcg groups. The proportion of opioid-free patients at 24 and 48 h decreased with decreasing dose of ITM; 41% in 150 mcg group, 31% in the 125 mcg group and 28% in the 100 mcg group at 24 h. At 48 h, this reduces to 24% in the 150 mcg group, 15% (37.5% reduction in the 125 mcg group, and 13% (45.8% reduction) in the 100 mcg group. This designates analgesic inferiority of both 125 mcg and 100 mcg ITM doses compared to 150 mcg dosing. There was no significant difference between 125 and 100 mcg dosing. The duration of analgesia was greater in 150 mcg dosing (median duration 21 h) compared to 125 mcg (13 h) and 100 mcg (12 h) groups. No significant difference in pain scores was noted between doses. FAS scores demonstrated a trend towards functional limitation in 125 mcg and 100 mcg dosing compared to 150 mcg. There was no difference between the 125 mcg and 100 mcg groups. Increased pruritus was seen in the 150 mcg and 125 mcg groups (41%) compared to the 100 mcg group (32%). Increased nausea and vomiting was seen in the 150 mcg (49%) and 125 mcg (42%) groups compared to the 100 mcg group (24%). No difference in LOS was noted between doses (median difference 1.1 h).

**Conclusion:**

All doses of ITM provide effective analgesia with 100 mcg dosing providing the best trade-off between analgesic efficacy and side effect profile.

## Introduction

In recent years, Enhanced Recovery Protocols have made their way into obstetric practice with demonstrated benefits including, reduced length of hospital stay (LOS) and opioid consumption (Crandon et al. 2024; Ljungqvist et al. 2017; Cavallaro and Bordeianou 2019). Despite significant variations in enhanced recovery protocols, a consistent component is multimodal analgesia. Neuraxial anaesthesia with Bupivacaine and Fentanyl is the most common and effective anaesthetic approach for facilitation of caesarean section (Gomez et al. 2020; Weigl et al. 2017). A popular enhancement is the inclusion of intrathecal morphine (ITM) to this combination, which has been found to act in a complementary manner, increasing the quality of intra-operative analgesia and the duration of post-operative analgesia (Weigl et al. 2017; Lui et al. 2017). ITM was first described for obstetric analgesia in the early 1980 s, and its inclusion is now considered the ‘gold standard’ providing analgesia for up to 24 h (Sultan et al. 2016). Its use is strongly endorsed by the Society for Obstetric Anaesthesia and Perinatology (SOAP), and as such is included in their updated Enhanced Recovery After Caesarean Section (ERAC) consensus statement and recommendations (Bollag et al. 2021). The use of ITM is designed to reduce the need for long-acting opioids, improve patient function, and increase the number of patients who are opioid free on discharge (Crandon et al. 2024).

Although the use of ITM is well established, the recommended dose it yet to be defined. An online survey from the SOAP demonstrated that 87% of Anaesthetist’s use between 50 and 250 mcg of ITM (Sultan et al. 2016). This wide range in dosing occurs due to a dose dependent ‘trade-off’ between analgesic efficacy and the incidence of side effects (Sharpe et al. 2020; Crandon et al. 2024). Early dosing studies with ITM demonstrated the possibility of a ceiling effect at 75–100 mcg for analgesia above which there was no significant improvement in pain or reduction in rescue opioids. However, there was noted a sharp and significant increase in pruritus and nausea and vomiting (Palmer et al. 1999; Girgin et al. 2008). A later study by Wong et al. in 2013, evaluating this topic, found that the previous studies may have been underpowered and unable to detect a change in opioid consumption for lower ITM dose ranges, despite a trend towards needing increased rescue opioids (Wong et al. 2013). Wong et al., conducted their own study powered to detect changes in opioid requirements and found a 19% reduction in opioid consumption at 24 h in patients receiving 200 mcg ITM (44 mg ± 35 mg oral morphine equivalents), compared to those who received 100 mcg (54 mg ± 35 mg oral morphine equivalents) (Wong et al. 2013). The study also reinforced a significant increase in pruritus and nausea and vomiting at 200 mcg compared to 100 mcg. This leads to the question, what is more important, analgesia or avoidance of complications? Studies into this question have suggested that although pain after caesarean section is a highly undesirable outcome, nausea and vomiting may be nearly as feared by patients (Macario et al. 1999; Smith et al. 2021). If so, the increased analgesic benefit of the higher dose ITM may be potentially negated by increased nausea and vomiting in some populations (Macario et al. 1999; Carvalho et al. 2005).

### Aims

The primary aims were to compare and define the analgesic and side effect profile of intrathecal morphine within an already established ERAC protocol. Here, we assessed 150 mcg, 125 mcg, and 100 mcg dosing of ITM. This involved evaluating total oral opioid requirements for the first 24 and 48 h, measured in oral morphine equivalents (milligrams), and the proportion of opioid-free patients. The frequency of opioid-related side-effects, specifically, rates of nausea and vomiting and pruritus was also evaluated.

Secondary aims were to (1) evaluate post-operative pain at 24 h post-caesarean section. This was measured via numerical pain scores at rest and with movement and through functional activity score (FAS). (2) Duration of analgesia, measured from introduction of spinal anaesthesia to first rescue opioid (measured in hours) and (3) length of stay (LOS), measured in hours from time of spinal anaesthetic to discharge. We also assessed and compared major complication rates between groups, including medical emergency team (MET) calls and escalation of post-operative care to the high dependency unit (HDU) or intensive care unit (ICU) post caesarean section.

### Study population

The population was defined as females over 18 years of age, ASA 2, undergoing repeat, elective, and caesarean section. No primiparous women were included in this study for standardisation purposes.

## Methods

### Interventions

The ERAC interventions included the following:

- ∘ Intensive education for all clinical (anaesthetic and obstetric) and nursing (midwifery, theatre, and post-anaesthetic care unit (PACU) staff involved in patient care throughout the peri-operative journey.
- ∘ Pre-operative consultation with the obstetric clinical nurse consultant (CNC) which included a peri-operative education package for the patients, 1 week prior to their booked surgical date.
- ∘ Pre-operative carbohydrate drink, taken the morning of surgery.
- ∘ Post-operative vital sign monitoring (hourly for the first 12 h post-operatively, followed by 2-hourly for the following 12 h).
- ∘ Post-operative analgesics, anti-emetics, anti-pruritics, to be prescribed by the anaesthetic team guided by a standardised Electronic Patient Record (iEMR) ‘PowerPlan’ for ITM use in caesarean section (approved by the safety committee).
- ∘ All women offered multimodal analgesia with rescue doses of oral analgesia available on the ward, consisting of, regular oral paracetamol and ibuprofen, with tramadol and oxycodone available PRN.
- ∘ Day 1 post-operative review by the obstetric CNC, which included a post-operative cognitive aid checklist for patients to ensure safety prior to discharge.
- ∘ ITM dosing
  - ∘ ITM dose is the only intervention of the ERAC protocol that was adjusted between groups
  - ∘ Patients received either 150 mcg, 125 mcg, or 100 mcg of ITM

## Study design, sample population, and statistical analysis

This study was a single centre, prospective cohort study, set as a ‘non-inferiority’ design, involving 573 women (190 in the 150 mcg group, 191 in the 125 mcg group and 193 in the 100 mcg group) undergoing elective caesarean section. After ethical approval from the Hospital and Health Service Human Research Ethics Committee, patients meeting inclusion criteria were identified and approached to participate in the study. Patients with significant medical history or obstetric comorbidities (defined as ASA 3 or greater), history of chronic pain, body mass index (BMI) over 35 and those requiring a general anaesthetic or emergency caesarean section and those who were not repeat caesarean sections (e.g. primiparous women) were excluded from the study. Informed consent was obtained from all women participating.

A total of 573 women were prospectively recruited between 16 February 2021 and 30 October 2022. All patients were recruited consecutively in blocks for each dosing category. Potential participants underwent consultation by the obstetric CNC 1 week prior to the day of surgery and if eligible were enrolled and consented, highlighted on the Electronic Patient Record and flagged to the anaesthetist on the day of surgery. All patients received standard ERAC care, with the only change between patients being the ITM dose. A spinal anaesthetic with heavy bupivacaine, 15 mcg fentanyl and ITM was given to each patient. Data was first collected for the 150 mcg group, followed by the 125 mcg then 100 mcg groups. The time-period continued until the required sample size was achieved in each group.

The primary outcome measures were total oral opioid requirements for the first 24 and 48 h (converted to milligrams of oral morphine equivalents), the proportion of opioid-free patients (at 24 and 48 h) and frequency of ITM-related side-effects (pruritus and nausea and vomiting).

Secondary outcome measures included duration of analgesia (measured via a surrogate, hours to first rescue opioid), post-operative pain scores at 24 h, post-operative FAS at 24 h, LOS (hours from administration of spinal anaesthetic to discharge) and frequency of serious peri-operative complications (respiratory depression, medical emergency team (MET) calls and escalation to HDU or ICU.

## Statistical analysis

In a previous study by Crandon et al., when intrathecal morphine was given at a dose of 150 μg, 41% of patients remained opioid free in the first 24 h post-caesarean section. However, nearly 50% of patients experienced nausea and/or vomiting and nearly 42% of patients experienced pruritus. By decreasing the dose of ITM to 125 or 100 mcg we hope to decrease the proportion experiencing nausea and vomiting by a clinically significant margin (absolute reduction of 20% as determined by consensus of clinical authors and colleagues) from 50 to 30%, and a similar absolute reduction for pruritus. At the same time, we considered a decrease from 41 to 32.8% (relative reduction of 20% or absolute difference of 8.2%) for those not requiring oral opioids at 24 h to be an acceptable trade off.

An a priori sample size estimate indicated that 93 participants would be required in each group for a non-inferiority margin of 8.2%, with 80% power at the 5% significance level (G*power: one-sided proportions test). Due to the nature of our recruitment process, the time-period each ITM dose was in use resulted in between 190 and 193 participants being recruited to each group. This provided a power of 98% to detect an improvement of 50% to 30% for nausea and vomiting.

Logistic regression (and the proportions test) was used to test differences between proportions experiencing nausea and vomiting, pruritus, receiving oral morphine within 24 or 48 h, or FAS A status, depending on the ITM dose used. While no predefined significance level was set, the Marascuilo procedure for multiple comparisons of proportions was used to provide a conservative benchmark for the significance of the *P*-value.

Length of stay in hours from time of birth to discharge was compared between ITM dose groups. Normality was assessed by visual inspection of histograms and by the Shapiro–Wilk test. As the distribution could not be transformed to normality, distributions were described by the median and interquartile range and the Kruskal–Wallis test with post hoc Dunn test (Sidak adjustment for multiple comparisons) were used for comparisons. Quantile (median) regression was also used to assess the effect of ITM dose. Similar analyses were performed for pain scores (ordinal data) at 24 h.

A time to event analysis was performed to determine the effects of ITM dose on time to first post-operative oral opioid. Kaplan–Meier survival curves were constructed and Cox proportional hazards analyses performed and the proportional hazards assumption tested. Median times to first oral opioid were also calculated for each ITM dose group.

## Results

### Opioid consumption

Comparison of total opioid consumption was made between groups at 24 and 48 h. We assessed the overall oral morphine equivalents as well as the proportion of patients that were opioid-free at 24 and 48 h. The median opioid consumption at 24 h and 48 h is shown in Table 1. The proportion of opioid free patients for each dose group at 24 and 48 h is shown in Table 2.

**Table 1:** Summary of oral morphine equivalents at 24 and 48 h

Dose	150 mcg	125 mcg	100 mcg	*P*-value
Sample size (*n*)	190	191	193	
Median (IQR) mg 24 h	10 (0, 27.5)	20 (0, 40)	20 (0, 37.5)	0.006
Mean (SD) mg 24 h	16.9 (21.0)	23.1 (23.0)	22.4 (22.5)	
Median (IQR) mg 48 h	30 (7.5, 67.5)	45 (17.5, 87.5)	45 (20, 80)	0.003
Mean (SD) mg 48 h	41.2 (42.3)	52.8 (42.5)	50.9 (41.3)	

*n* number in group, *IQR* interquartile range, *SD* standard deviation, *P*-value from Kruskal-Wallis test (Dunn tests: 24 h: 150v125, *P* = 0.005; 150v100, *P* = 0.01, 125v100, *P* = 0.79; 48 h: 150v125, *P* = 0.003; 150v100, *P* = 0.009, 125v100, *P* = 0.72)

**Table 2:** Summary of proportion of opioid free patients

	150 mcg	125 mcg	100 mcg	*P*-value
Sample size (*n*)	190	191	193	
% opioid free 24 h (95% CI)	41% (34, 48)	31% (25, 38)	29% (23, 35)	0.01
% opioid free 48 h (95% CI)	24% (18, 31)	15% (11, 21)	14% (9, 19)	0.006

*n* number in group, *95% CI* 95% confidence interval, *P* value from Cochran-Armitage test for trend. Pairwise comparisons (logistic regression): 24 h: 150v125, *P* = 0.04; 150v100, *P* = 0.01, 125v100, *P* = 0.6; 48 h: 150v125, *P* = 0.03; 150v100, *P* = 0.008, 125v100, *P* = 0.6.

*P* < 0.05 after multiple comparison adjustment

**Fig. 1:**
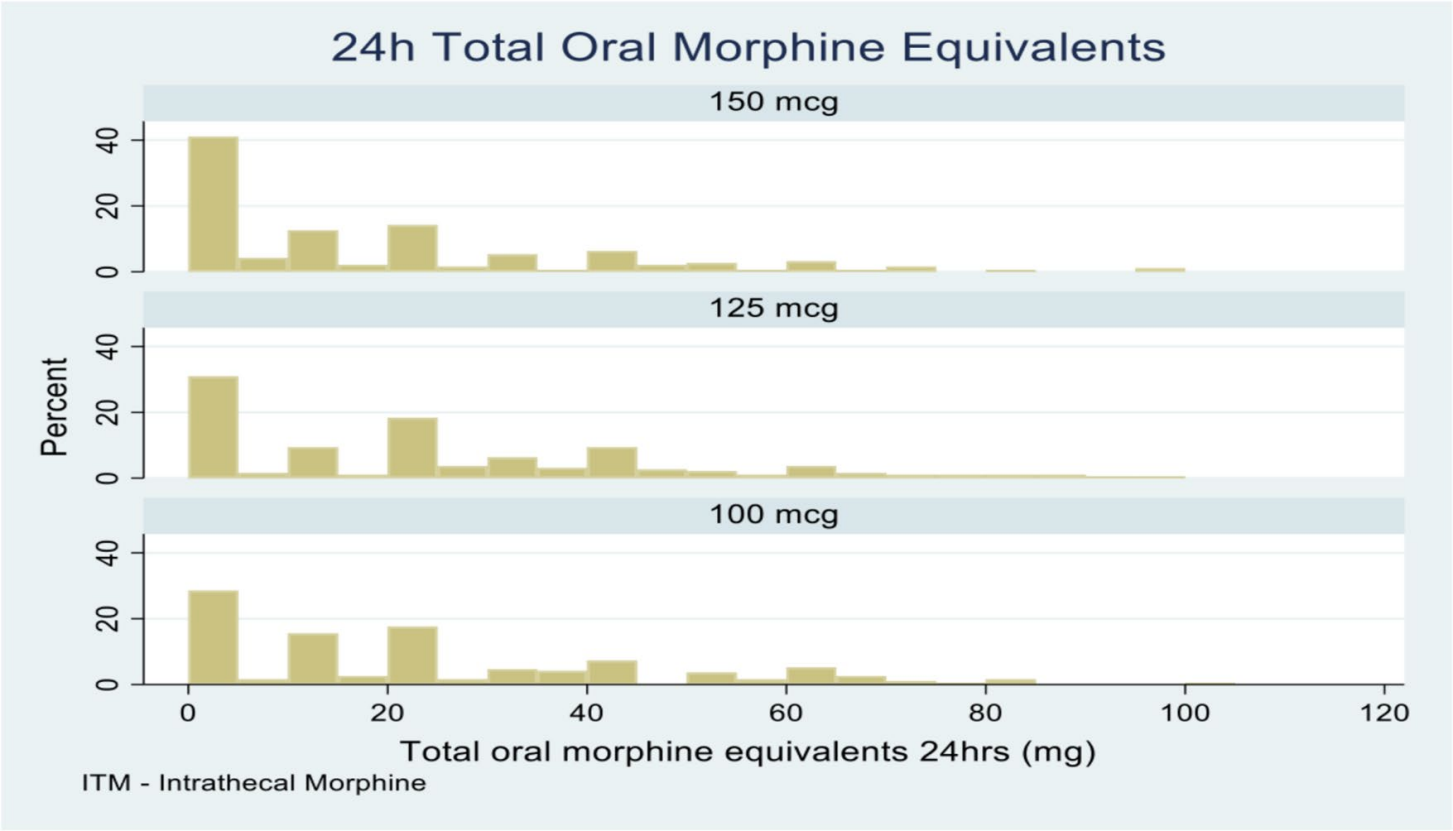
Histogram of total oral morphine equivalents at 24 h

Figure 1 is a histogram demonstrating a trend for increasing need for rescue opioids with decreasing dose of intrathecal morphine in the first 24 h post-delivery. 41% of patients were opioid free in the 150 mcg group at 24 h. This reduced to 31% and 28% of patients in the 125 mcg and 100 mcg groups.

**Fig. 2:**
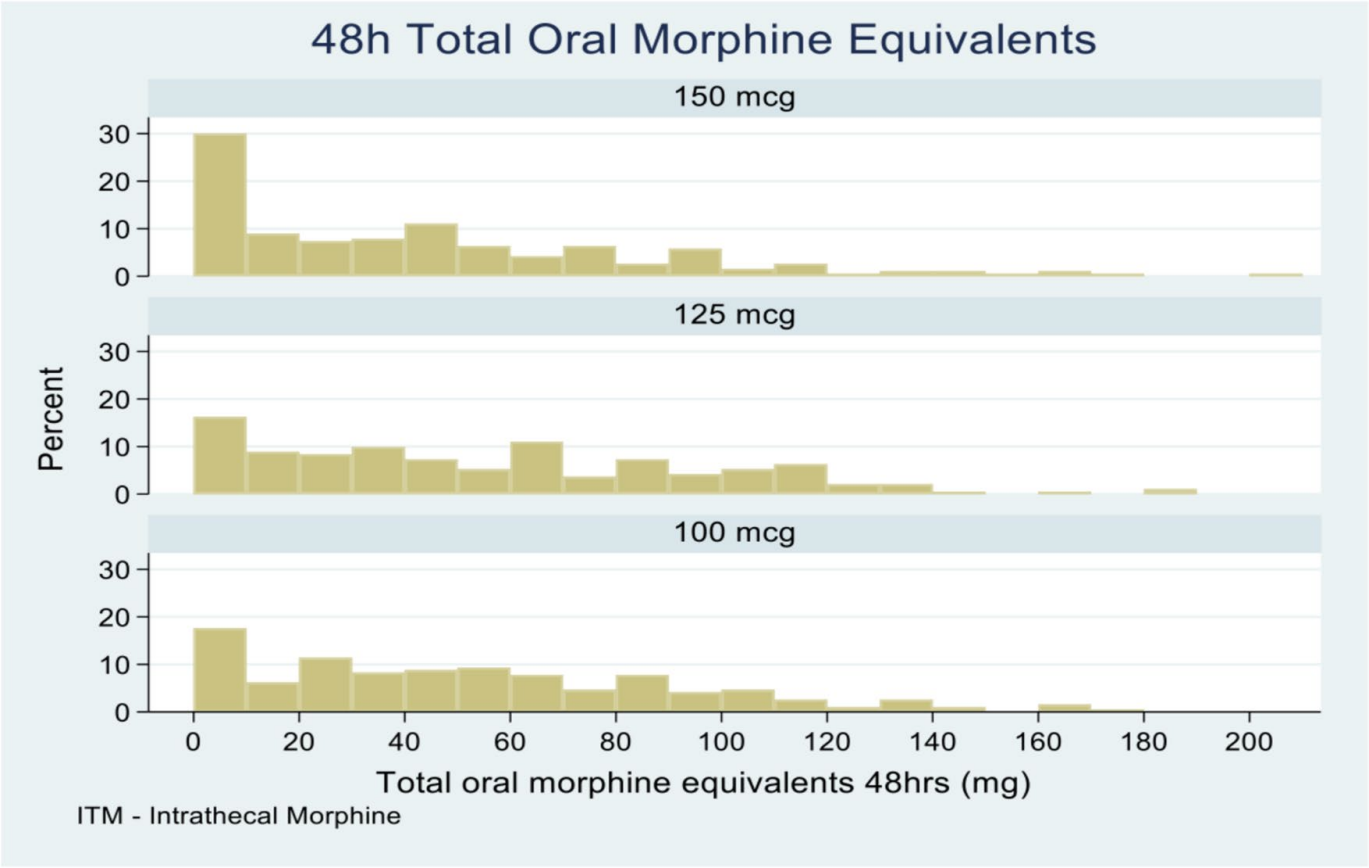
Histogram of total oral morphine equivalents at 48 h

Figure 2 is a histogram demonstrating a trend for increased oral opioid requirements with decreasing intrathecal morphine dosing in the first 48 h. At 48 h, 24% of patients were opioid free in the 150 mcg group. This reduced to 15% and 13% in the 125 mcg and 100 mcg groups.

### Pruritus

The frequency of pruritus was assessed between ITM dosing groups. Forty-one percent of patients in the 150 mcg and 125 mcg group and 32% of patients in the 100 mcg group reported pruritus. In the 150 mcg group, 24 patients (13%) received Naloxone and 28 patients (15%) received antihistamines. Twenty-five patients (13%) in 125 mcg group received Naloxone and 19 patients (10%) received antihistamines. Twenty-two patients (11%) in the 100 mcg group were given Naloxone and 17 patients (9%) received antihistamines. No Naloxone infusions or refractory symptoms were encountered in any group (Table 3).

**Table 3:** Summary of pruritus and treatment proportions

	150 mcg	125 mcg	100 mcg
Sample size (*n*)	190	191	193
Pruritus %	41% (79)	41% (79)	32% (62)
Antihistamine PRN	15% (28)	10% (19)	9% (17)
Naloxone PRN	13% (24)	13% (25)	11% (22)
Refractory pruritus	0% (0)	0% (0)	0% (0)

Summary of the proportions of patients experiencing pruritus for each dosing group, and treatments used. *P*-value 0.07 (150 mcg v. 100 mcg and 125 mcg v. 100 mcg)

A Pearson’s chi-squared test, Fisher exact test, and subsequently a two-sample test of proportions was performed. Despite a trend demonstrating decreased pruritus in the 100 mcg ITM group, this was not statistically significant (*P*-value 0.07). This however denotes a 22% reduction in pruritus in the 100 mcg ITM group compared to both the 150 mcg and 125 mcg groups.

### Nausea and vomiting

The frequency of nausea and vomiting was analysed between groups. Forty-nine percent (93) of patients in the 150 mcg group reported nausea and vomiting. All except 1 patient reported alleviation of symptoms with simple antiemetics. In the 125 mcg group, 42% (80) patients reported nausea and vomiting requiring treatment. Two patients reported refractory symptoms in this group despite simple antiemetics. In the 100 mcg group, 24% of patients reported nausea and vomiting. All patients in this group responded to simple antiemetics with no refractory symptoms reported.

The data between groups was analysed using a chi-squared and Fisher’s exact test demonstrating a significant difference between groups, with the primary difference being accounted for through the 100 mcg group. A two-sample test of proportions was then conducted, demonstrating a statistically significant difference between both 150 mcg and 125 mcg compared to the 100 mcg group. Meaning there is both a clinically and statistically significant reduction in nausea and vomiting in the 100 mcg ITM group compared to 150 mcg and 125 mcg groups (Table 4).

**Table 4:** Summary of nausea and vomiting proportions

	150 mcg	125 mcg	100 mcg
Sample size (*n*)	190	191	193
Nausea and vomiting %	49% (93)	42% (80)	24% (46)
Resolved with 3 antiemetics	99% (92)	98% (78)	100% (46)
Refractory nausea and vomiting	0.5% (1)	1% (2)	0 (0%)

Proportion of patients experiencing nausea and vomiting for each dosing group. *P*-value 0.20 (150 mcg v. 125 mcg), < 0.0001 (150 mcg v. 100 mcg), < 0.0002 (125 mcg vs 100 mcg)

### Duration of analgesia

**Fig. 3:**
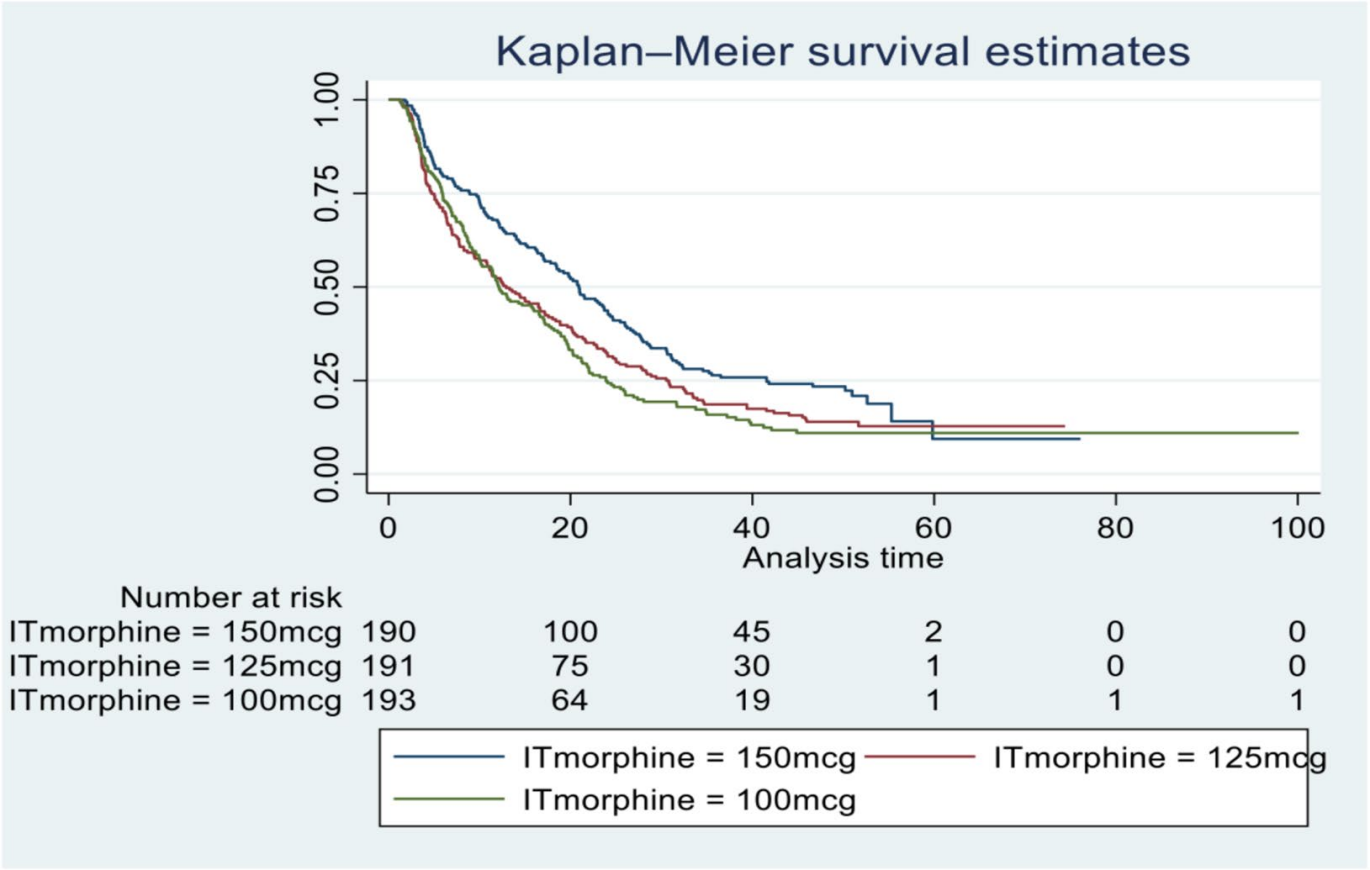
Time to event analysis for first rescue opioid use

The duration of action of each dose-level of ITM was assessed using a surrogate, the time until the first rescue opioid was required (Table 5). The proportion not requiring opioids and the median time to first rescue opioid are shown for each group. LOS is also shown to demonstrate that non-requirement of opioids was not an artifact of early censoring (loss to follow-up). A time to event analysis (first rescue opioid) was performed with Kaplan–Meier survival curves, shown in Fig. 3, and hazard ratio estimates in Table 5.

**Table 5:** Summary of time to first rescue opioid

	150 mcg	125 mcg	100 mcg
Proportion not requiring opioids during LOS (*n*); LOS median (IQR)	21.6% (41); 49.6 (45.5, 51.5)	14.7% (28); 50.4 (46.1, 53.5)	13.4% (26); 46.6 (28.7, 50.5)
Time to 1 st opioid (hours); median (IQR); (*n*); LOS median (IQR)	15.3 (5.8, 24.7); 149; 51.3 (49.1, 54.3)	10.0 (4.1, 21.6); 163; 52.2 (49.6, 57.5)	10.1 (5.4, 19.6); 167; 51.9 (49.5, 57.5)
Hazard ratio (95% CI) vs 150 mcg	–	1.36 (1.09, 1.69)	1.51 (1.21, 1.88)
Hazard ratio 100 mcg vs 125 mcg (95% CI)			1.11 (0.90, 1.38)

Comparison between curves: 150 mcg v. 125 mcg -HR = 1.36, *P* = 0.007; 150 mcg v. 100 mcg -HR = 1.51, *P* = 0.0003; 125 mcg v. 100 mcg -HR = 1.1, *P* = 0.34

### Pain scores

Pain scores, using the numerical scale (0 to 10), were evaluated at 24 h, both at rest and with movement for each dose group. Pain scores were not recorded for 12 patients (6%) in the 150 mcg group, 11 patients (5.7%) in the 125 mcg group and 7 patients (3.6%) in the 100 mcg group. Median pain scores at rest were the same for all groups (median of 1). With movement, the median pain scores were 2 for the 150 mcg and 125 mcg groups and 3 for the 100 mcg group. A Kruskal–Wallis and Dunn test was performed. At rest, there was no significant difference in pain scores between dosing groups. With movement, no statistically significant difference was identified between the 100 mcg group and 150 mcg (0.05). There was also no statistical difference identified between the 150 mcg and 125 mcg groups nor the 125 mcg and 100 mcg groups (Table 6).

**Table 6:** Statistical difference

	150 mcg	125 mcg	100 mcg
Sample size (*n*)	178	179	186
median (rest)	1	1	1
Mean (rest)	1.5	1.6	1.6
Median movement	2	2	3
Mean (movement)	2.4	2.5	2.8

**Fig. 4:**
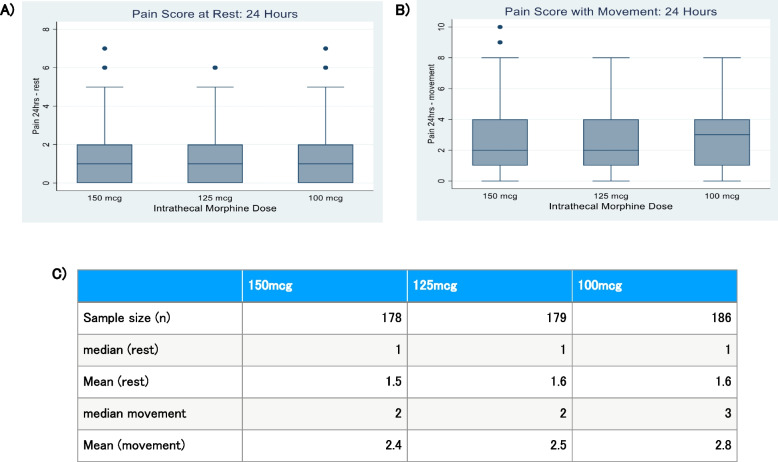
Summary statistics for pain scores at 24 h

Figure 4 shows box plot of pain scores (A) at rest for each dosing group, and (B) with movement in the first 24 h. Below are the *P*-values for comparison between groups. (C) Summary table for pain score statistic. *P*-value at rest, 0.12 (150 mcg v. 125 mcg and 100 mcg v. 125 mcg), 0.48 (150 mcg v. 100 mcg). *P*-value with movement, 0.25 (150 mcg v. 125 mcg), 0.05 (150 mcg v. 100 mcg), 0.15 (125 mcg v. 100 mcg).

### Functional assessment scores (FAS)

Functional Assessment Scores were compared between groups at 24 h. All groups demonstrated a predominance towards FAS A with 91% in the 150 mcg group, 83% in the 125 mcg group and 79.3% in the 100 mcg group. A trend towards increasing FAS B frequency was observed as ITM dose decreased. No FAS C were recorded in any group.

Chi-squared analyses was performed, and a difference was found between groups (*P*-value 0.005). Logistic regression was used to assess for significance between proportions of FAS scores for each dosing group. Statistical significance was identified between the 150 mcg and 125 mcg groups (*P*-value = 0.025) and between the 150 mcg and 100 mcg groups (*P*-value = 0.002). There was no statistically significant difference between the 125 mcg and 100 mcg groups (*P*-value = 0.32) (Table 7).

**Table 7:** Summary of FAS results at 24 h

	150 mcg	125 mcg	100 mcg
FAS A	173 (91.1%)	159 (83%)	153 (79.3)
FAS B	17 (8.9%)	32 (16.8%)	40 (21%)
FAS C	0 (0%)	0 (0%)	0 (0%)
Total	190 (100%)	191 (100%)	193 (100%)

Summary of proportions of each category of FAS score for each dosing group at 24 h. *P*-value 0.025 (150 mcg v. 125 mcg), 0.002 (150 mcg v. 100 mcg), 0.32 (125 mcg v. 100 mcg)

### Length of stay

A Kruskal–Wallis test followed by Dunn test was performed to assess for differences between groups (Table 8). All groups were noted to form a large cluster around the 50-h mark with 82% of patients in the 150 mcg ITM group, 80% of the 125 mcg group and 78% of those in the 100 mcg group being discharged prior to 55 h.

**Table 8:** Length of stay

Dose (*n*)	150 mcg (190)	125 mcg (191)	100 mcg (193)	*P*-value
Median (IQR) hours	50.8 (48.6, 53.6)	51.9 (49.4, 54.9)	51.6 (49.0, 55.6)	0.06

*n* number in group, *IQR* interquartile range, *P*-value from Kruskal-Wallis test (Dunn tests: 150v125, *P* = 0.03; 150v100, *P* = 0.14, 125v100, *P* = 0.60)

**Fig. 5:**
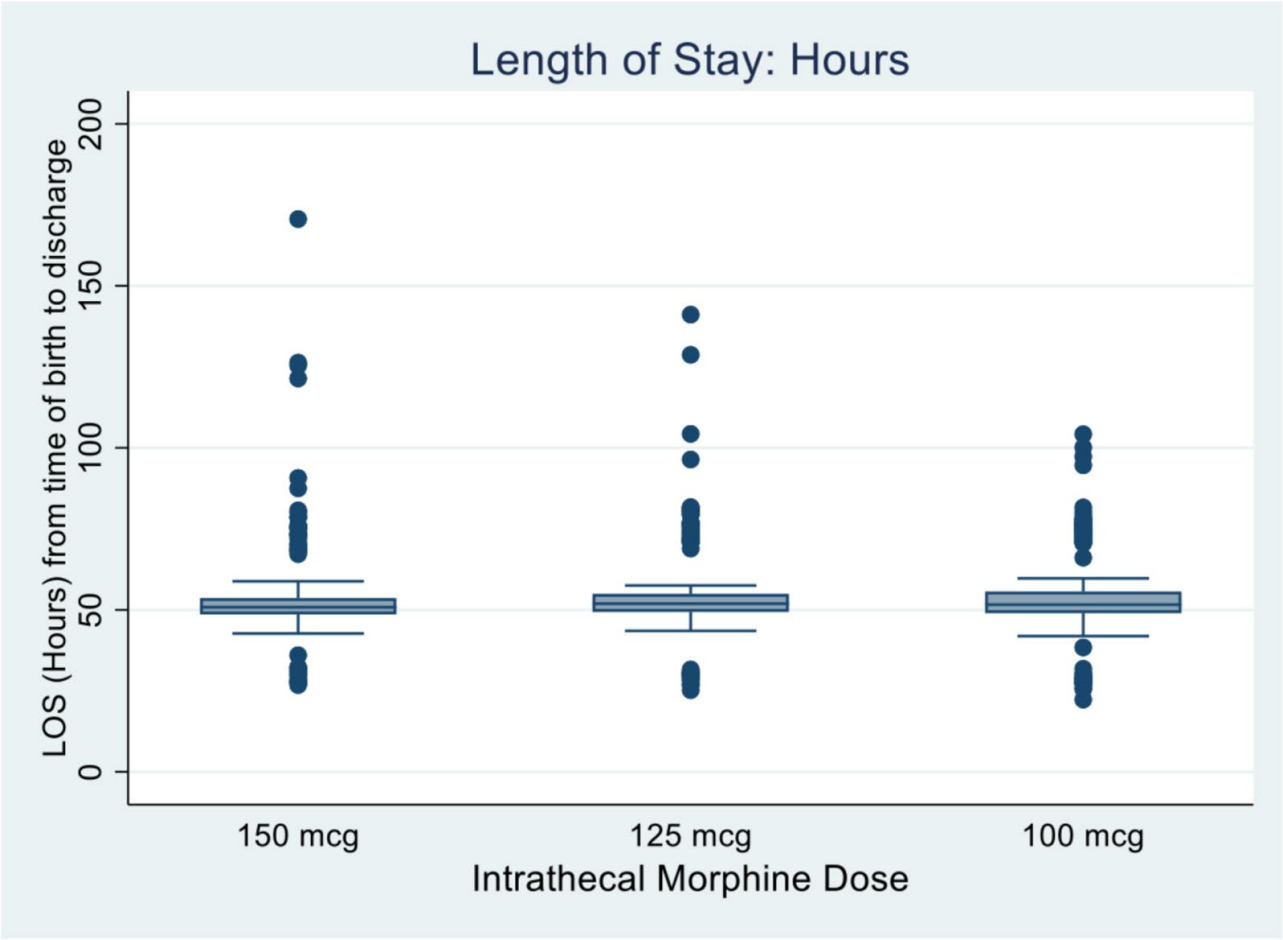
Box plot length of stay (hours)

Figure 5 represents a box plot of the length of stay in hours for each of the three groups. All groups cluster around the 50-h mark. There is no significant clinical difference between groups, meaning that a dose reduction of intrathecal morphine from 150 to 125 mcg and 100 mcg does not significantly alter the length of stay for patients.

### Other complications

There were no cases of respiratory depression reported in any group. There was no emergency calls or escalation to ICU for patients in any groups.

## Discussion

This study has demonstrated that intrathecal morphine (ITM) doses between 100 mcg and 150 mcg provide effective analgesia following caesarean section. Reducing the ITM dose from 150 to 100 mcg resulted in a mild increase in opioid consumption, but significantly improved the side-effect profile. These findings are consistent with previous literature demonstrating a dose-dependent increase in the frequency of side effects (Crandon et al. 2024; Palmer et al., 1999; Girgin et al., 2008; Wong and Carvalho,  2013).

Further evidence supporting effective analgesia across all doses is reflected in the overall clinically small doses of rescue oral opioid consumed at both 24 and 48 h. When compared to studies not using ITM, oral opioid consumption is significantly greater. In a recent study by Crandon et al. (2024), a median oral morphine equivalent (OME) of 120 mg at 24 h (IQR 90–145 mg) and 177.5 mg at 48 h (IQR 132.5–222.5 mg) was seen in the group not given ITM. This is significantly greater than the rescue opioid doses across all study groups, and is a reflection of effective analgesia at all ITM doses. Median OME consumption was 10 mg at 24 h and 30 mg at 48 h in the 150 mcg group, compared to 20 mg and 45 mg, respectively, in the 125 mcg and 100 mcg groups. Although statistically significant, the maximum median difference between groups—10 mg at 24 h and 15 mg at 48 h—is unlikely to be clinically meaningful.

There was a statistically significant reduction in the proportion of opioid-free patients with decreasing ITM doses. This parameter was used to assess potential analgesic inferiority. Due to a lack of established clinical thresholds in the literature, the authors agreed upon a conservative criterion: a ≥ 20% reduction in the proportion of opioid-free patients between groups would indicate inferiority. Based on this, the 150 mcg group had 41% of patients opioid-free at 24 h, compared to 31% (24.4% reduction) in the 125 mcg group and 28% (31.7% reduction) in the 100 mcg group. At 48 h, opioid-free rates were 24% in the 150 mcg group, versus 15% (37.5% reduction) and 13% (45.8% reduction) in the 125 mcg and 100 mcg groups, respectively. These results suggest that 125 mcg and 100 mcg doses are inferior to 150 mcg based on our predefined criteria. However, no statistical difference was observed between the 125 mcg and 100 mcg groups, indicating that 100 mcg is not inferior to 125 mcg.

Consistent with existing literature, our data have confirmed a dose-dependent increase in ITM side effects (Armstrong and Fernando, 2016; Palmer et al., 1999; Girgin et al., 2008; Wong and Carvalho, 2013). Pruritus was reported in 41% of patients receiving 150 mcg or 125 mcg of ITM, compared to 32% in the 100 mcg group—a 22% relative reduction. Although not statistically significant (*P* = 0.07), this may still be clinically relevant, given the high frequency of pruritus post-ITM and the high use of treatments like naloxone or antihistamines (approximately 60% across all groups). No cases of refractory pruritus were noted.

Nausea and vomiting, which are among the most distressing postoperative side effects (Macario et al., 1999; Carvalho et al. 2005), also followed a dose-dependent pattern: 49% of patients in the 150 mcg group, 42% in the 125 mcg group, and 24% in the 100 mcg group. The difference between the 100 mcg group and both higher-dose groups was statistically significant (*P* < 0.0001 vs. 150 mcg; *P* = 0.0002 vs. 125 mcg). No significant difference was found between the 150 mcg and 125 mcg groups (*P* = 0.20). Refractory nausea occurred in one patient in the 150 mcg group and two in the 125 mcg group.

Analgesic duration was assessed using ‘time to first opioid’ administration as a surrogate marker. The assumption being a shorter duration of action would be reflected by earlier need for rescue opioids. Previous studies suggest a dose-dependent relationship in ITM duration (Sultan et al., 2016; Sultan and Carvalho 2021). Our findings support this. The 150 mcg group had a median time to first opioid of 21 h, significantly longer than the 125 mcg group (13 h) and 100 mcg group (12 h). This demonstrates that patients receiving 100 mcg required rescue opioids approximately 9 h earlier than those given 150 mcg.

Using this data, a Kaplan–Meier time-to-event curve was constructed, allowing visualisation of time to first opioid and calculation of hazard ratios. Statistically significant differences were observed between the 150 mcg group and both the 125 mcg (*P* = 0.007) and 100 mcg (*P* = 0.001) groups. No significant difference was found between the 125 mcg and 100 mcg groups (*P* = 0.33). The 150 mcg group consistently showed a higher proportion of opioid-free patients over time. Hazard ratios revealed that patients in the 100 mcg group were 1.5 times (50%) more likely to require a rescue opioid at any given time compared to the 150 mcg group; for the 125 mcg group, this increased likelihood was 1.3 times (30%). These findings further support a dose-dependent relationship in ITM duration of action.

Caesarean section pain is often described as moderate-to-severe and can impair a mother’s ability to care for her newborn (Gamez and Habib, 2018; Weibel et al. 2016; Gerbershagen et al. 2013). It is therefore reassuring that reducing ITM from 150 mcg to 100 mcg did not significantly affect pain scores, which remained in the mild range across all groups, both at rest and with movement.

Functional Assessment Scores (FAS) at 24 h revealed most patients experienced no pain-related functional limitations. Specifically, 91% of patients in the 150 mcg group, 83% in the 125 mcg group, and 79% in the 100 mcg group were classified as FAS A (no limitation). However, a trend towards increased FAS B scores (mild limitation) was observed with lower ITM doses: 9% in the 150 mcg group, increasing to 16.8% in the 125 mcg group and 21% in the 100 mcg group. These differences were statistically significant between 150 mcg and both 125 mcg (*P* = 0.025) and 100 mcg (*P* = 0.002), but not between 125 and 100 mcg (*P* = 0.32). Despite this, no FAS C scores (inability to perform activities due to pain) were recorded.

There was no significant difference in hospital length of stay between groups. The largest median difference was just 1.1 h (50.8 h for 150 mcg vs. 51.9 h for 125 mcg; 51.6 h for 100 mcg). This finding has implications for hospital resource planning and cost-effectiveness, showing that lower ITM doses do not extend hospitalisation.

No serious complications, such as respiratory depression, medical emergency team (MET) calls, or ICU escalation, were reported in any group, indicating a high degree of safety across all dosing levels.

Despite its strengths, this study has limitations. First, the study design would have benefited from being a randomised controlled trial (RCT), which would have allowed for randomisation and blinding of both patients and clinicians, thereby reducing potential bias. However, logistical and resource constraints prevented this. Second, the collection of FAS data only at 24 h limited assessment of longer-term functional outcomes. Extending this data collection to 48 h could have provided valuable insight into whether trends in functional limitation persisted beyond the period of ITM activity.

## Conclusion

This study offers valuable insights into the analgesic efficacy and side-effect profile of intrathecal morphine in obstetric anaesthesia. All doses tested (100–150 mcg) were found to be safe and effective for post-caesarean section analgesia. Reducing the dose from 150 to 100 mcg resulted in minimal increase in opioid consumption, with no significant change in pain scores or hospital length of stay. However, lower doses significantly improved the side-effect profile.

Given this balance between effective analgesia and reduced side effects, we recommend 100 mcg of ITM as the optimal dose for caesarean section. It offers adequate pain relief, a reduced incidence of adverse effects, and does not increase resource use—making it a pragmatic and patient-centred choice in clinical practice. We do however feel patient discussion is needed and assessment of their concerns and goals prior to deciding on a dosing strategy.

## Data Availability

No datasets were generated or analysed during the current study.
